# FMixFN: A Fast Big Data-Oriented Genomic Selection Model Based on an Iterative Conditional Expectation algorithm

**DOI:** 10.3389/fgene.2021.721600

**Published:** 2021-11-18

**Authors:** Wenwu Xu, Xiaodong Liu, Mingfu Liao, Shijun Xiao, Min Zheng, Tianxiong Yao, Zuoquan Chen, Lusheng Huang, Zhiyan Zhang

**Affiliations:** State Key Laboratory for Pig Genetic Improvement and Production Technology, Jiangxi Agricultural University, Nanchang, China

**Keywords:** genomic selection, model, big data-oriented, GEBV, FMixFN

## Abstract

Genomic selection is an approach to select elite breeding stock based on the use of dense genetic markers and that has led to the development of various models to derive a predictive equation. However, the current genomic selection software faces several issues such as low prediction accuracy, low computational efficiency, or an inability to handle large-scale sample data. We report the development of a genomic prediction model named FMixFN with four zero-mean normal distributions as the prior distributions to optimize the predictive ability and computing efficiency. The variance of the prior distributions in our model is precisely determined based on an F2 population, and genomic estimated breeding values (GEBV) can be obtained accurately and quickly in combination with an iterative conditional expectation algorithm. We demonstrated that FMixFN improves computational efficiency and predictive ability compared to other methods, such as GBLUP, SSgblup, MIX, BayesR, BayesA, and BayesB. Most importantly, FMixFN may handle large-scale sample data, and thus should be able to meet the needs of large breeding companies or combined breeding schedules. Our study developed a Bayes genomic selection model called FMixFN, which combines stable predictive ability and high computational efficiency, and is a big data-oriented genomic selection model that has potential in the future. The FMixFN method can be freely accessed at https://zenodo.org/record/5560913 (DOI: 10.5281/zenodo.5560913).

## Introduction

Based on the use of genomic information and prediction of the genetic merit of animals, genomic selection is changing breeding strategies and approaches in livestock (Goddard and Hayes, 2009). Among many agricultural animals and plants, estimated breeding values (EBV) predicted from genomic information are now widely used (Duchemin et al., 2012; Pollak et al., 2012; Preisinger, 2012; Ibáñez-Escriche et al., 2014; Samorè and Fontanesi, 2016; Mrode et al., 2018). Comparative studies on both simulated and real data have shown that genomic EBV (GEBV) tends to have higher accuracy than breeding values estimated using pedigree relationships. The accuracy of GEBV is mainly impacted by the nature of the single nucleotide polymorphism (SNP) panel used, the size of the training data, the population structure, the relationships between individuals in the training and validation population, and the genetic architecture of the trait, in particular, the number of loci affecting the trait and the distribution of their effects (Daetwyler et al., 2008; Goddard, 2009; Meuwissen, 2009). Usually, the most accurate method to predict genetic value or phenotype based on the SNP genotypes is to fit all SNPs simultaneously, treating the SNP effects as they are drawn from a prior distribution that matches as closely as possible the true distribution of SNP effects (Goddard, 2009; Chatterjee et al., 2013). The assumption that, in the SNP-best linear unbiased prediction (BLUP) or genomic BLUP (GBLUP) model, each of the SNPs explains equal variance, i.e., that the more complex traits are controlled by very many quantitative trait loci (QTL), each with a tiny effect, could be imprecise if a trait is affected by a small number of QTL, each with a large effect (Meuwissen et al., 2001; VanRaden, 2008). In other models, the distribution of SNP effects is allowed to depart from a pseudo-infinitesimal distribution. BayesA extended the SNP-BLUP model by estimating the variance of each marker separately, and an inverse chi-square prior was used to estimate these variances (Meuwissen et al., 2001). In BayesB, it was assumed that most of the markers have a zero effect on the targeted trait, and the prior distribution of the variances is a mixture of a distribution with zero variance and an inverse chi-squared distribution, with some SNPs having a zero effect, and some SNPs having a large effect on the trait (Meuwissen et al., 2001). The true distribution of the effect sizes is not known, but a mixture of normal distributions can approximate a wide variety of distributions by varying the mixing proportions (Mclachlan Basford. et al., 1988; Silverman, 1996; Luan et al., 2009; Moser et al., 2015; Goddard et al., 2016). Kemper et al. and Erbe et al. presented and extended a model named “BayesR,” which used a mixture of four normal distributions as prior, each with a zero mean but with variances of 0, 0.0001 $\delta_{g}^{2}$ , 0.001 $\delta_{g}^{2}$ , and 0.01 $\delta_{g}^{2}$ for genomic prediction (Erbe et al., 2012; Kemper et al., 2015). In the applications of this model, it has been assumed that the mixing proportions are drawn from a Dirichlet distribution with parameters (1, 1, 1, 1). In a simulation study in which the genetic model included a finite number of loci with exponentially distributed effects, the Bayes-based model provided more accurate prediction of genetic value than GBLUP.

Although Bayes-based models have the potential for the development of more faithful genetic models and seem to be the best choice for estimating GEBV, they require long computing times since they use computer-intensive MCMC techniques (Meuwissen et al., 2001; Xu, 2003; Verbyla et al., 2010; Habier et al., 2011; Cheng et al., 2015). For practical applications and for computer simulations of genomic selection breeding schemes, which need many selection rounds and replications, it would be useful to have a much faster algorithm for the calculation of Bayes-based GEBV. Several non-MCMC algorithms have been proposed to improve computational efficiency for linear models with differential shrinkage of SNP effects or with variable selection. Methods BL and BhGLM were developed by Xu et al. and Yi et al., respectively, which used Expectation-Maximization (EM) algorithms (Yi and Banerjee, 2009; Xu, 2010). VanRaden et al. (2009) presented two non-linear predictions A and B that are analogous to the BayesA and BayesB, respectively. Meuwissen et al. (2009) presented a fast heuristic iterative conditional expectation (Zhao et al.) algorithm, where the posterior expectation of SNP effects was calculated analytically, assuming a fixed known double exponential (DE) parameter and dispersion parameters. Dong et al. (2017) formulated an algorithm based on the same model as the ICE algorithm, which uses a product of univariate densities instead of the multivariate normal density to estimate SNP effects, but the a priori hypothesis on the size of the SNP effects is based on the Pareto principle, which was proposed by the economist Vilfredo Pareto at the beginning of the 20th century. This principle states that approximately 20% of the population possesses 80% of the wealth in a country. Similar theories have been further applied in various fields, such as in genomic prediction by Yu and Meuwissen (2011). In their study, the a priori distribution of the genomic prediction model was a mixture of two normal distributions, which assumes that x% of the SNPs explain (100 − x)% of the genetic variance, and the remaining  (100 − x)% of SNPs explain the remaining x% of genetic variance (Grosfeld-Nir et al., 2007). Using this economic principle to assume the a priori distribution of the marker effect is not very convincing, leading to only a general predictive accuracy in Dong’s research.

For genomic selection, most of the focus has been on prediction accuracy and computational efficiency, but the computing limits are an increasingly important aspect that needs to be taken into account. The direct method of genomic selection can provide GEBV in a short computing time when the number of individuals in the population is small, but some studies have shown that when the dimensions of the kinship matrix exceed hundreds of thousands or even millions, the process to inverse the matrix inverse becomes very difficult due to the limitations in computer memory and computational time (Misztal, 2016). According to the Council on Dairy Cattle Breeding (https://queries.uscdcb.com/Genotype/cur_freq.html), more than 5,000,000 Holstein cows have been genotyped as of July 2021. With the accumulation of breeding data, there is an urgent need for a genomic selection model that can handle large-scale sample data.

In our study, we presented an ICE-based prediction model with four zero-mean normal distributions as the prior distribution and the variance of which have been obtained accurately based on the 374 standardized phenotypes in an F2 population. This model with four normal distributions and variances classified into four categories was referred to as FMixFN, where MixFN refers to the prior distribution of FMixFN was a mixture of four normal distributions, and the first “F” refers to “Fast.” As a test, the predictive ability and computation time obtained with GBLUP, SSgblup, Bayesian mixture regression (MIX), BayesR, BayesA, BayesB, and FMixFN were compared first based on six traits with different heritabilities and different genetic architectures by using cross-validation. Then FMixFN was evaluated by using data from Duroc and Asian rice experiments, respectively (Zhao et al., 2011; Ding et al., 2019). This study also evaluated the efficiency of FMixFN and its ability to handle large-scale sample data with 20 sets of sample data simulated by the QMsim software.

## Materials and Methods

All procedures including experimental animals established and tissue collection were performed in accordance with the guidelines approved by the Ministry of Agriculture of China. This study was approved by the ethics committee of Jiangxi Agricultural University.

### Data

An F2 design resource population was developed between 2000 and 2006 (Guo et al., 2009) as follows: two White Duroc sires and 17 Erhualian dams were mated to produce F1 animals, from which 9 F1 boars and 59 F1 sows were intercrossed (avoiding full-sib mating) to produce 967 F2 males and 945 F2 females (in total *n* = 1,912) in six batches. All the F2 animals were kept under standard indoor conditions at the experimental farm of Jiangxi Agricultural University (China). Then the F2 piglets were weaned at 46 days, and males were castrated at 90 days. At 240 ± 6 days of age, 1,030 F2 animals including 549 gilts and 481 barrows were slaughtered at 70–120 kg live weight.

Genomic DNA was isolated from ear tissue with a standard phenol/chloroform extraction method. All DNA samples were diluted to a final concentration of 50 ng/μl in 96-well plates. In total, 933 F2 were genotyped with the Illumina PorcineSNP60 BeadChip on an iScan System (Illumina, United States) following the manufacturer’s protocol (Ramos et al., 2009). Quality control procedures were implemented by PLINK (version 1.07) (Chang et al., 2015). Briefly, SNPs with unspecific positions on the genome build 10.2, a call rate lower than 90%, and a minor allele frequency (MAF) lower than 1% were eliminated, and animals with a missing typing rate higher than 10% were also removed. In total, 374 phenotypes were measured on the individuals of the F2 population, including carcass traits, reproductive traits, immune traits, meat traits, growth traits, and epigenetic traits (see Additional file 1: Supplementary Table S1). These 374 traits were then divided into three groups according to their heritability, i.e., 68 traits with high heritability ( $h^{2}>0.4$ ), 148 traits with a moderate heritability ( $0.2<h^{2}<0.4$ ), and 158 with low heritability ( $h^{2}<0.2$ ).

### Estimation of Substitution Effects

We used the GEMMA software to calculate the substitution effects of 14,320,159 SNPs on the 374 traits included in the standard linear model (Zhou and Stephens, 2012). Sex was included as a fixed effect, and heritability was estimated by using the −lmm procedure implemented in GEMMA. Population stratification was adjusted by including a genomic relationship matrix. Briefly, the model was as follows:

$$y=Wa+X\beta +u+e\;;u∼MVN_{n}\left(0,\sigma_{u}^{2}K\right),e∼MVN_{n}\left(0,\sigma_{e}^{2}I_{n}\right)$$

where y is an n element vector of phenotypic values, all the traits were normalized before calculation so that the substitution effects were comparable among all the phenotypes, W is a design matrix of covariates, a is a vector of fixed effects, X is a vector of genotypes at each locus, β is the effect size of SNPs, and u is the vector of random effects following a multivariate normal distribution $MVN_{n}\left(0,\sigma_{u}^{2}K\right)$ , e is the vector of errors following $MVN_{n}\left(0,\sigma_{e}^{2}I_{n}\right)$ , $\sigma_{u}^{2}$ and $\sigma_{e}^{2}$ are polygenic variance and residual variance, respectively, which are estimated based on the REML average information (AI) algorithm. K is a known kinship matrix estimated from genome sequence variants, and $I_{n}$ being an n×n identity matrix.

### Distribution of Additive Genetic Variance

Three genotypes “AA,” “Aa,” and “aa” were assumed each locus and were represented by 0, 1, and 2, respectively, with p and q the frequencies of alleles “A” and “a,” respectively. Assuming that the effect value of this locus is estimated as β (with no dominance), the additive genetic variance can be expressed as $2pq\beta^{2}$ (Park et al., 2011). In the group of traits with high heritability, all the loci for each phenotype were put together and ranked by additive genetic variance from large to small. And all the ordered loci were equally divided into four groups. For each group, the proportion of the sum of the additive genetic variances of all loci to the total additive genetic variance (or variance-ratio thereafter) was calculated, equal to a1 , b1 , c1 , and d1 , respectively (Subsequently called variance ratio). Similarly, the same method was used for the groups of traits with a moderate heritability and a low heritability, resulting in a2 , b2 , c2 , d2 , and a3 , b3 , c3 , d3 , respectively. Therefore, the four expected variances in each of the three groups can be expressed as:

Group of traits with high heritability:

$$\;\delta_{1}^{2}=\frac{a1V_{g}}{\gamma M};\delta_{2}^{2}=\frac{b1V_{g}}{\gamma M};\delta_{3}^{2}=\frac{c1V_{g}}{\gamma M};\delta_{4}^{2}=\frac{d1V_{g}}{\gamma M}$$

Group of traits with a moderate heritability:

$$\;\delta_{1}^{2}=\frac{a2V_{g}}{\gamma M};\delta_{2}^{2}=\frac{b2V_{g}}{\gamma M};\delta_{3}^{2}=\frac{c2V_{g}}{\gamma M};\delta_{4}^{2}=\frac{d2V_{g}}{\gamma M}$$

Group of traits with a low heritability:

$$\;\delta_{1}^{2}=\frac{a3V_{g}}{\gamma M};\delta_{2}^{2}=\frac{b3V_{g}}{\gamma M};\delta_{3}^{2}=\frac{c3V_{g}}{\gamma M};\delta_{4}^{2}=\frac{d3V_{g}}{\gamma M}$$

Where $V_{g}$ and M is the additive variance and the number of markers, respectively. γ is set to 0.25.

### Analytical Derivation for FMixFN

The linear model for genomic prediction was as follows:

y=Xb+Bg+e

Where n individuals and m SNPs were assumed. Thus, y is a n×1 vector of phenotypes recorded; b is the vector of fixed effects; g is a m×1 vector of additive SNP effects; e is a vector of residual errors; X is the design matrix for fixed effects; and B is standardized design matrix for additive SNP effects (coded as 0 for genotype “AA,” 1 for “Aa” and 2 for “aa,” respectively).

In this study, the prior distribution with four zero-mean normal distributions was written as a function of prior distributions of SNP variance as determined above:

$$\pi \left(g_{j}\right)=\;\gamma \phi \left(g_{j}\text{|}0,\delta_{1}^{2}\right)+\gamma \phi \left(g_{j}\text{|}0,\delta_{2}^{2}\right)+\gamma \phi \left(g_{j}\text{|}0,\delta_{3}^{2}\right)+\gamma \phi \left(g_{j}\text{|}0,\delta_{4}^{2}\right);\gamma =0.25$$ (1)

$\pi \left(g_{j}\right)$ is the univariate normal distribution, the effects of SNPs are obtained by using the Iterative Conditional Expectation algorithm (Meuwissen et al., 2009). In brief, assume that $E\left(g_{j}\text{|}y_{-j}\right)$ is estimated, the current effects of all the other SNPs are used to calculate the $y_{-j}$ as follows:

$$y_{-j}=y-Xb-\underset{k\neq j}{\sum }B_{k}g_{k}$$

where $B_{k}$ is a vector from the $k^{th}$ column of B, the expectation of SNP effect, $E\left(g_{j}\text{|}y_{-j}\right)$ , is then estimated by a Bayesian model in the next round:

$$E\left(g_{j}\text{|}y_{-j}\right)=\int_{-\infty }^{+\infty }g_{j}f\left(g_{j}\text{|}y_{-j}\right)dg_{j}$$

$$=\frac{\int_{-\infty }^{+\infty }g_{j}f\left(y_{-j}|B_{j}g_{j},I\delta_{e}^{2}\right)\pi \left(g_{j}\right)dg_{j}}{f\left(y_{-j}\right)}$$

$$=\frac{\int_{-\infty }^{+\infty }g_{j}f\left(y_{-j}|B_{j}g_{j},I\delta_{e}^{2}\right)\pi \left(g_{j}\right)dg_{j}}{\int_{-\infty }^{+\infty }f\left(y_{-j}|B_{j}g_{j},I\delta_{e}^{2}\right)\pi \left(g_{j}\right)dg_{j}}$$ (2)

$f\left(y_{-j}\right)$ is a marginal distribution function of $y_{-j}$ and can be calculated using the law of total cumulance: $\int_{-\infty }^{+\infty }f\left(y_{-j}|B_{j}g_{j},I\delta_{e}^{2}\right)\pi \left(g_{j}\right)dg_{j}$ . Calculating $f\left(y_{-j}|B_{j}g_{j},I\delta_{e}^{2}\right)$ is computationally demanding because it is a multivariate normal density, which involves calculating the determinant and the inverse of the variance-covariance matrix for the data $y_{-j}$ . Therefore, we simplified the derivation by using a univariate normal densities $\;f\left(Y|g_{j},\delta^{2}\right)$ to replace $f\left(y_{-j}|B_{j}g_{j},I\delta_{e}^{2}\right)$ , where $Y={\left(B_{j}^{'}B_{j}\right)}^{-1}B_{j}^{'}y_{-j}$ and $\delta^{2}={\left(B_{j}^{'}B_{j}\right)}^{-1}\delta_{e}^{2}$ ; details of the derivation process are as follows:

$$\begin{array}{l} f\left(y_{-j}\text{|}B_{j}g_{j},I\delta_{e}^{2}\right)\propto exp\left[-\frac{{\left(y_{-j}-B_{j}g_{j}\right)}^{,}\left(y_{-j}-B_{j}g_{j}\right)}{2\delta_{e}^{2}}\right]\\ =\exp\left(-\frac{y_{-j}^{,}y_{-j}-2B_{j}^{,}y_{-j}g_{j}+B_{j}^{,}B_{j}g_{j}^{2}}{2\delta_{e}^{2}}\right)\\ =\exp\left[-\frac{g_{j}^{2}-2{\left(B_{j}^{,}B_{j}\right)}^{-1}B_{j}^{,}y_{-j}g_{j}+{\left(B_{j}^{,}B_{j}\right)}^{-1}y_{j}^{,}y_{-j}}{2{\left(B_{j}^{,}B_{j}\right)}^{-1}\delta_{e}^{2}}\right]\\ =exp\left\{-\frac{{\left[g_{j}-{\left(B_{j}^{,}B_{j}\right)}^{-1}B_{j}^{,}y_{-j}\right]}^{2}-{\left[{\left(B_{j}^{,}B_{j}\right)}^{-1}B_{j}^{,}y_{-j}\right]}^{2}+{\left(B_{j}^{,}B_{j}\right)}^{-1}y_{j}^{,}y_{-j}}{2{\left(B_{j}^{,}B_{j}\right)}^{-1}\delta_{e}^{2}}\right\} \end{array}$$

$$\propto exp\left\{-\frac{{\left[g_{j}-{\left(B_{j}^{,}B_{j}\right)}^{-1}B_{j}^{,}y_{-j}\right]}^{2}}{2{\left(B_{j}^{,}B_{j}\right)}^{-1}\delta_{e}^{2}}\right\}\propto exp\left[-\frac{{\left(g_{j}-Y\right)}^{2}}{2\delta^{2}}\right]\propto f\left(Y|g_{j},\delta^{2}\right)$$

Therefore, the equation $E\left(g_{j}\text{|}y_{-j}\right)$ can be written as:

$$E\left(g_{j}\text{|}y_{-j}\right)=\frac{\int_{-\infty }^{+\infty }g_{j}f\left(Y|g_{j},\delta^{2}\right)\pi \left(g_{j}\right)dg_{j}}{\int_{-\infty }^{+\infty }f\left(Y|g_{j},\delta^{2}\right)\pi \left(g_{j}\right)dg_{j}}$$ (3)

the numerator of Eq. 3 can be broken down into four terms combined with Eq. 1 as follows:

$$\gamma \int_{-\infty }^{+\infty }g_{j}f\left(Y\text{|}g_{j},\delta^{2}\right)\phi \left(g_{j}\text{|}0,\delta_{1}^{2}\right)dg_{j}$$

$$+\gamma \int_{-\infty }^{+\infty }g_{j}f\left(Y\text{|}g_{j},\delta^{2}\right)\phi \left(g_{j}\text{|}0,\delta_{2}^{2}\right)dg_{j}$$

$$+\gamma \int_{-\infty }^{+\infty }g_{j}f\left(Y\text{|}g_{j},\delta^{2}\right)\phi \left(g_{j}\text{|}0,\delta_{3}^{2}\right)dg_{j}$$

$$+\gamma \int_{-\infty }^{+\infty }g_{j}f\left(Y\text{|}g_{j},\delta^{2}\right)\phi \left(g_{j}\text{|}0,\delta_{4}^{2}\right)dg_{j}$$ (4)

The first term of Eq. 4 can be derived as follows:

$$\begin{array}{l} \gamma \int_{-\infty }^{+\infty }g_{j}f\left(Y\text{|}g_{j},\delta^{2}\right)\phi \left(g_{j}\text{|}0,\delta_{1}^{2}\right)dg_{j}\\ =\;\gamma \int_{-\infty }^{+\infty }g_{j}\frac{1}{\delta \sqrt{2\pi }}exp\left[-\frac{{\left(Y-g_{j}\right)}^{2}}{2\delta^{2}}\right]\frac{1}{\delta_{1}\sqrt{2\pi }}exp\left[-\frac{g_{j}^{2}}{2\delta_{1}^{2}}\right]dg_{j}\\ =\frac{\gamma }{\sqrt{2\pi }}\int_{-\infty }^{+\infty }\frac{g_{j}}{\delta \delta_{1}\sqrt{2\pi }}exp\left[-\frac{{\left(Y-g_{j}\right)}^{2}}{2\delta^{2}}-\frac{g_{j}^{2}}{2\delta_{1}^{2}}\right]dg_{j}\\ =\frac{\gamma }{\sqrt{2\pi }}\int_{-\infty }^{+\infty }\frac{g_{j}}{\delta \delta_{1}\sqrt{2\pi }}exp\left[-\frac{\left(g_{j}^{2}-\frac{2Y\delta_{1}^{2}}{\delta^{2}+\delta_{1}^{2}}g_{j}+\frac{Y^{2}\delta_{1}^{2}}{\delta^{2}+\delta_{1}^{2}}\right)\left(\delta^{2}+\delta_{1}^{2}\right)}{2\delta^{2}\delta_{1}^{2}}\right]dg_{j}\\ =\;\frac{\gamma }{\sqrt{2\pi }}\int_{-\infty }^{+\infty }\frac{g_{j}}{\delta \delta_{1}\sqrt{2\pi }}exp\left[-\frac{{\left(g_{j}-\frac{Y\delta_{1}^{2}}{\delta^{2}+\delta_{1}^{2}}\right)}^{2}\left(\delta^{2}+\delta_{1}^{2}\right)}{2\delta^{2}\delta_{1}^{2}}-\frac{Y^{2}}{2\left(\delta^{2}+\delta_{1}^{2}\right)}\right]dg_{j}\\ =\frac{\gamma }{\sqrt{2\pi }}exp\left[-\frac{Y^{2}}{2\left(\delta^{2}+\delta_{1}^{2}\right)}\right]\frac{1}{\sqrt{\delta^{2}+\delta_{1}^{2}}}\int_{-\infty }^{+\infty }\frac{g_{j}}{\frac{\delta \delta_{1}}{\sqrt{\delta^{2}+\delta_{1}^{2}}}\sqrt{2\pi }}exp\left[-\frac{{\left(g_{j}-\frac{Y\delta_{1}^{2}}{\delta^{2}+\delta_{1}^{2}}\right)}^{2}}{2{\left(\frac{\delta \delta_{1}}{\sqrt{\delta^{2}+\delta_{1}^{2}}}\right)}^{2}}\right]dg_{j} \end{array}$$

Here, the last term of this formula equals $\frac{Y\delta_{1}^{2}}{\delta^{2}+\delta_{1}^{2}}$ as it can be taken as calculating the expected value of $g_{j}$ in the normal distribution with mean $\frac{Y\delta_{1}^{2}}{\delta^{2}+\delta_{1}^{2}}$ , and variance $\frac{\delta^{2}\delta_{1}^{2}}{\delta^{2}+\delta_{1}^{2}}$ . Therefore, the first term of Eq. 4 can be written as follows:

$$\frac{\gamma }{\sqrt{2\pi }}\exp\left[-\frac{Y^{2}}{2\left(\delta^{2}+\delta_{1}^{2}\right)}\right]\frac{1}{\sqrt{\delta^{2}+\delta_{1}^{2}}}\frac{Y\delta_{1}^{2}}{\delta^{2}+\delta_{1}^{2}}$$

Here, the derivation process for the remaining terms of Eq. 4 was the same as for this term, and therefore, the final form of the numerator of Eq. 3 is:

$$\frac{\gamma }{\sqrt{2\pi }}\exp\left[-\frac{Y^{2}}{2\left(\delta^{2}+\delta_{1}^{2}\right)}\right]\frac{1}{\sqrt{\delta^{2}+\delta_{1}^{2}}}\frac{Y\delta_{1}^{2}}{\delta^{2}+\delta_{1}^{2}}$$

$$+\frac{\gamma }{\sqrt{2\pi }}\exp\left[-\frac{Y^{2}}{2\left(\delta^{2}+\delta_{2}^{2}\right)}\right]\frac{1}{\sqrt{\delta^{2}+\delta_{2}^{2}}}\frac{Y\delta_{2}^{2}}{\delta^{2}+\delta_{2}^{2}}$$

$$+\frac{\gamma }{\sqrt{2\pi }}\exp\left[-\frac{Y^{2}}{2\left(\delta^{2}+\delta_{3}^{2}\right)}\right]\frac{1}{\sqrt{\delta^{2}+\delta_{3}^{2}}}\frac{Y\delta_{3}^{2}}{\delta^{2}+\delta_{3}^{2}}$$

$$+\frac{\gamma }{\sqrt{2\pi }}\exp\left[-\frac{Y^{2}}{2\left(\delta^{2}+\delta_{4}^{2}\right)}\right]\frac{1}{\sqrt{\delta^{2}+\delta_{4}^{2}}}\frac{Y\delta_{4}^{2}}{\delta^{2}+\delta_{4}^{2}}$$ (5)

there is no $g_{j}$ in the integrand of the denominator in Eq. 3 compared to that of the numerator. Therefore, it should calculate the cumulative probability from −∞ to +∞ , but not calculate the expected value, and this value is 1. Thus, the denominator in Eq. 3 can be written as:

$$\frac{\gamma }{\sqrt{2\pi }}\exp\left[-\frac{Y^{2}}{2\left(\delta^{2}+\delta_{1}^{2}\right)}\right]\frac{1}{\sqrt{\delta^{2}+\delta_{1}^{2}}}$$

$$+\frac{\gamma }{\sqrt{2\pi }}\exp\left[-\frac{Y^{2}}{2\left(\delta^{2}+\delta_{2}^{2}\right)}\right]\frac{1}{\sqrt{\delta^{2}+\delta_{2}^{2}}}$$

$$+\frac{\gamma }{\sqrt{2\pi }}\exp\left[-\frac{Y^{2}}{2\left(\delta^{2}+\delta_{3}^{2}\right)}\right]\frac{1}{\sqrt{\delta^{2}+\delta_{3}^{2}}}$$

$$+\frac{\gamma }{\sqrt{2\pi }}exp\left[-\frac{Y^{2}}{2\left(\delta^{2}+\delta_{4}^{2}\right)}\right]\frac{1}{\sqrt{\delta^{2}+\delta_{4}^{2}}}$$ (6)

thus, the final form for Eq. 3 is derived:

$$E\left(g_{j}y_{-j}\right)=\frac{\frac{Y^{2}}{\delta^{2}+\delta_{1}^{2}}+\exp\left[\frac{Y^{2}}{2\left(\delta^{2}+\delta_{1}^{2}\right)}-\frac{Y^{2}}{2\left(\delta^{2}+\delta_{2}^{2}\right)}\right]\frac{\sqrt{\delta^{2}+\delta_{1}^{2}}}{\sqrt{\delta^{2}+\delta_{2}^{2}}}\frac{Y\delta_{2}^{2}}{\delta^{2}+\delta_{2}^{2}}+\exp\left[\frac{Y^{2}}{\left(\delta^{2}+\delta_{1}^{2}\right)}-\frac{Y^{2}}{\left(\delta^{2}+\delta_{3}^{2}\right)}\right]\frac{\sqrt{\delta^{2}+\delta_{1}^{2}}}{\delta^{2}+\delta_{3}^{2}}\frac{Y\delta_{3}^{2}}{\delta^{2}+\delta_{3}^{2}}+\exp\left[\frac{Y^{2}}{2\left(\delta^{2}+\delta_{1}^{2}\right)}-\frac{Y^{2}}{2\left(\delta^{2}+\delta_{4}^{2}\right)}\right]\frac{\sqrt{\delta^{2}+\delta_{1}^{2}}}{\delta^{2}+\delta_{4}^{2}}\frac{Y\delta_{4}^{2}}{\delta^{2}+\delta_{4}^{2}}}{1+\exp\left[\frac{Y^{2}}{2\left(\delta^{2}+\delta_{1}^{2}\right)}-\frac{Y^{2}}{2\left(\delta^{2}+\delta_{2}^{2}\right)}\right]\frac{\sqrt{\delta^{2}+\delta_{1}^{2}}}{\delta^{2}+\delta_{2}^{2}}+\exp\left[\frac{Y^{2}}{2\left(\delta^{2}+\delta_{1}^{2}\right)}-\frac{Y^{2}}{2\left(\delta^{2}+\delta_{3}^{2}\right)}\right]\frac{\sqrt{\delta^{2}+\delta_{1}^{2}}}{\delta^{2}+\delta_{3}^{2}}+\exp\left[\frac{Y^{2}}{2\left(\delta^{2}+\delta_{1}^{2}\right)}-\frac{Y^{2}}{2\left(\delta^{2}+\delta_{4}^{2}\right)}\right]\frac{\sqrt{\delta^{2}+\delta_{1}^{2}}}{\delta^{2}+\delta_{4}^{2}}}$$

Here, the fixed effects are estimated at each iteration by the formula: $\hat{b}={\left(X^{\prime }X\right)}^{-1}X^{\prime }\left(y-B\hat{g}\right)$ . Convergence of solutions at the t th iteration was judged based on the formula $\frac{{\left(G^{t}-G^{t-1}\right)}^{\prime }\left(G^{t}-G^{t-1}\right)}{{\left(G^{t}\right)}^{\prime }G^{t}}<10^{-8},$ where $G={\left({\hat{b}}^{'}{\hat{g}}^{\prime }\right)}^{\prime }$ . It ends at the iteration when all the SNPs have been calculated once.

### Analytical Models

In the following analysis, we used GBLUP (Meuwissen et al., 2001; VanRaden, 2008), SSgblup (Legarra et al., 2009; Christensen and Lund, 2010), FMixFN, MIX (Xavier et al., 2019), BayesR (Kemper et al., 2015), BayesA, and BayesB with respective model fittings to compare their performance, the variance components were pre-estimated using the mixed model. The details of these analyses were as follows:

GBLUP: GBLUP was used to estimate the effects of the markers by BLUP, assuming that each marker explains an equal proportion of the total genetic variance. The software GEMMA was used to implement the GBLUP calculation process (Zhou and Stephens, 2012).

SSgblup: Single-step genomic BLUP (SSgblup), which was developed by Aguilar et al. (2010) and Christensen and Lund (2010), opened the way to perform genomic prediction using phenotype, pedigree, and genomic information simultaneously on both genotyped and non-genotyped individuals via a combined relationship matrix (H). Implementation of SSgblup is completed by the R package “Hiblup” (https://hiblup.github.io/).

MIX: the MIXTURE model assumed that the marker effects came from a mixture of two distributions: one distribution with large variance (accommodating large marker effects) and one with small variance (accommodating small marker effects). The distribution to which the marker belongs is sampled from the Bernoulli distribution. The variances of the two distributions underlying the mixture are estimated using a noninformative chi-square distribution. Implementation of MIX is completed by the R package “VIGoR” (https://cran.r-project.org/web/packages/VIGoR/index.html).

BayesR: BayesR starts the hierarchical model and poses a mixture of four zero-mean normal distributions as a conditional prior for a specific SNP effect. We use BayesR software to implement the calculation process (https://cnsgenomics.com/software.html).

BayesA: BayesA assumes that the distribution of SNP effects follows a Student’s *t*-distribution. Mathematically, it is assumed that each SNP effect comes from a normal distribution but $\sigma^{2}$ can be varied among the SNPs because the *t*-distribution is not easy to incorporate into a prediction of the marker. A scaled inverted chi distribution, $X^{2}\left(\nu ,S\right)$ is usually used as prior for the variance components.

BayesB: The prior distribution of BayesB is a mixture distribution with some SNPs with zero effects and the rest with a *t*-distribution, and the prior hypothesis of the SNP with non-zero effect is the same as BayesA. The implementation of BayesA and BayesB is completed by the R package “BGLR” (Perez and de los Campos, 2014). All MCMC sampling was run for 50,000 cycles, and the first 20,000 cycles were discarded as burn-in for BayesR, BayesA, and BayesB.

### The Verification of Predictive Ability and Computing Time

To test the performance of FMixFN in terms of predictive ability and calculation time, we did the following. Firstly, the variance ratio of the prior distribution of FMixFN was assumed to be random, then two traits were selected to verify the unbiasedness, and the compatibility of the variance ratio estimated in the F2 population. The specific assumptions of the variance ratio are shown in Additional file 1: Supplementary Table S2, the first one is to average all variances, i.e. to set the classification with the largest variance ratio at 50%, and the second was to centralize all variances, i.e., the classification with the largest variance ratio is assumed to be 90%. Secondly, we compared the predictive ability and calculation time of FMixFN and other mainstream genomic selection methods and selected two phenotypes with different genetic structures and different heritabilities from each group for cross-validation, one trait is controlled by numerous polygenic genes and the other one is controlled by several loci with large variances. Supplementary Figure S1 (see Additional file 2: Supplementary Figure S1) shows that traits 1, 3, and 5 were controlled by SNPs with large variances, and traits 2, 4, and 6 were controlled by many SNPs, each with a very small effect. After quality control, the remaining number of individuals with the six traits were 839, 832, 834, 840, 784, and 838, respectively, with 33,901, 33,893, 33,891, 33,891, and 33,894 SNPs, respectively, the heritability of each trait was 0.600, 0.560, 0.380, 0.369, 0.145, and 0.107, respectively. Details on the number of individuals, number of SNPs, and heritability estimates are shown in Additional file 1: Supplementary Table S3. In addition, we also evaluated the stability of FMixFN by using data from the duroc experiment and Asian rice experiments, respectively, more specific information from this population is also shown in Additional file 1: Supplementary Table S3. Finally, to demonstrate that FMixFN can perform genomic prediction analysis based on large-scale sample data with no data overflow error, our study simulated 20 sets of sample data using QMsim software (Sargolzaei and Schenkel, 2009), which contains 10,000, 20,000, ……, 190,000, and 200,000 individuals, respectively. Each set of data was obtained through eight generations of mating, combining genotype and phenotype data from generations 3–8, and determining the number of individuals per generation by parameter setting. Genomic information of each individual was set with 10 chromosomes, each chromosome is set 100 cM long and including 101 markers and 100 QTLs, respectively, with a marker mutation rate of 2.5 and QTL mutation rate of 3. Genomic prediction by a replicated training-testing method was used to evaluate the predictive results. Cross-validation of nine replicates was performed. All individuals were randomly and evenly divided into nine groups. In each replicate, one of the groups was selected as the testing data set while the remaining eight groups were used as the training data set, and the results of each cross-validation are shown in Additional file 1: Supplementary Table S4. Predictive ability is defined as the correlation between GEBV and the phenotypes adjusted for the covariates $\left(y-X\hat{u}\right)$ (Meuwissen et al., 2001).

## Results

### The Expected Variance Ratio

In this study, all traits of the F2 population were divided into three groups based on the heritability of the traits: high, moderate, and low. For the group of traits with high heritability, the calculated a1 , b1 , c1 , and d1 were equal to 0.8752, 0.0958, 0.0256, and 0.0032, respectively. For the group of traits with a moderate heritability, the calculated a2 , b2 , c2 , and d2 were equal to 0.8367, 0.1246, 0.0342, and 0.0043, respectively. And for the group of traits with low heritability, the calculated a3 , b3 , c3 , and d3 were equal to 0.8225, 0.1413, 0.0324, and 0.0036, respectively. Those parameters were composed in the procedure of FMixFN, as FMixFN starts running, the program determines which group of variances is calculated based on the heritability of the experimental trait.

### Verification of Unbiasedness

In this study, we selected phenotype 3 and phenotype 4 to verify the unbiasedness of the variance ratio of the prior distribution. When the variance ratio was assumed to be 0.5, 0.25, 0.125, and 0.125, the predictive ability of phenotype 3 and phenotype 4 are 0.4773 and 0.4911, respectively. When the variance ratio is assumed to be 0.9, 0.005, 0.045, 0.005, the predictive ability of each of these phenotypes are 0.4789 and 0.4911, respectively. In contrast, the predictive ability of these two phenotypes estimated by using the original parameters is 0.4787 and 0.4913, respectively.

### Predictive Ability and Computing Time

**FIGURE 1:**
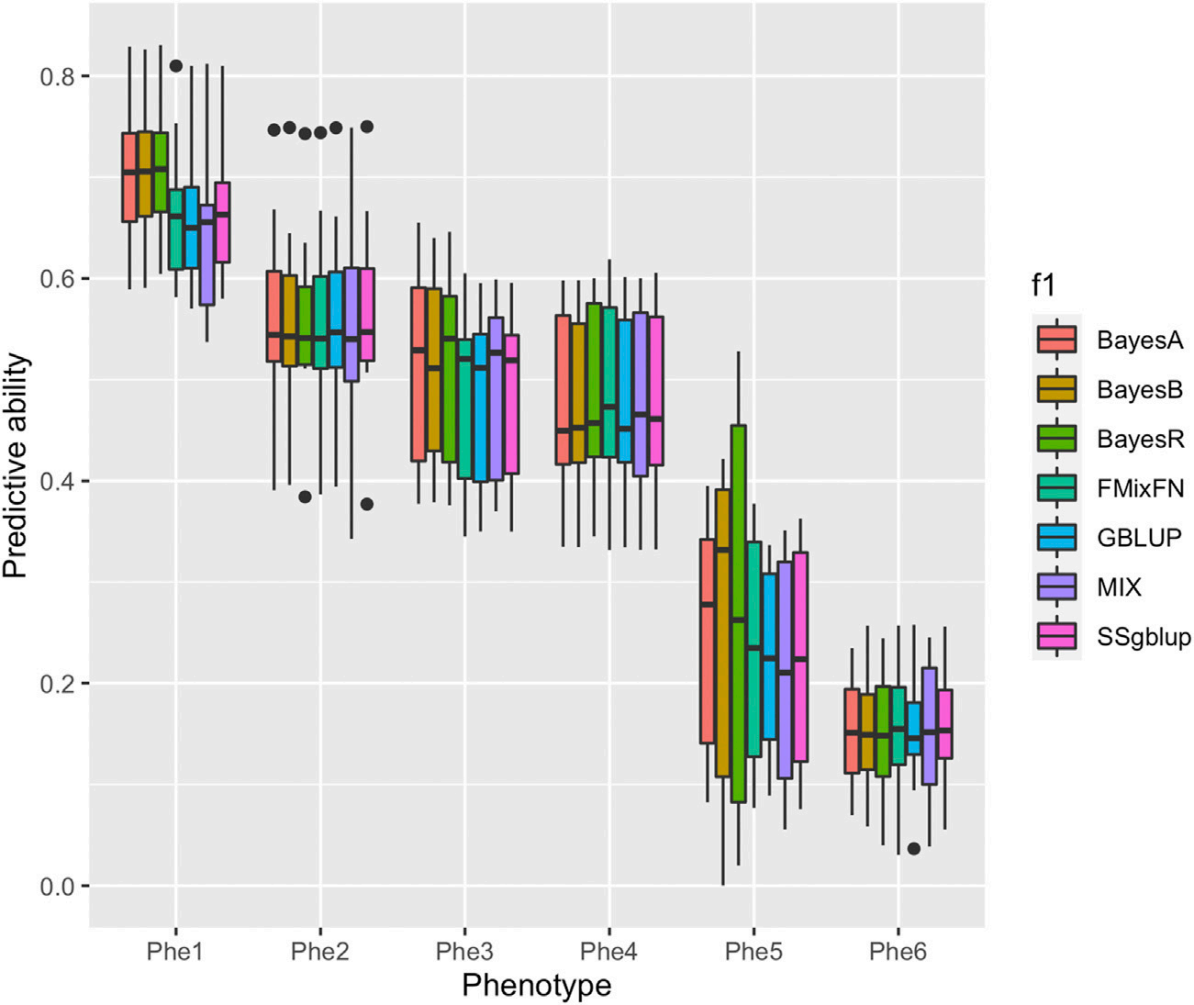
Comparison of predictive ability of BayesA, BayesB, BayesR, FMixFN, GBLUP, MIX, and SSgblup in all six traits. The predictive ability performance of each method was measured by the correlation method, which is the average Pearson correlation between predicted values and phenotypic values.

**FIGURE 2:**
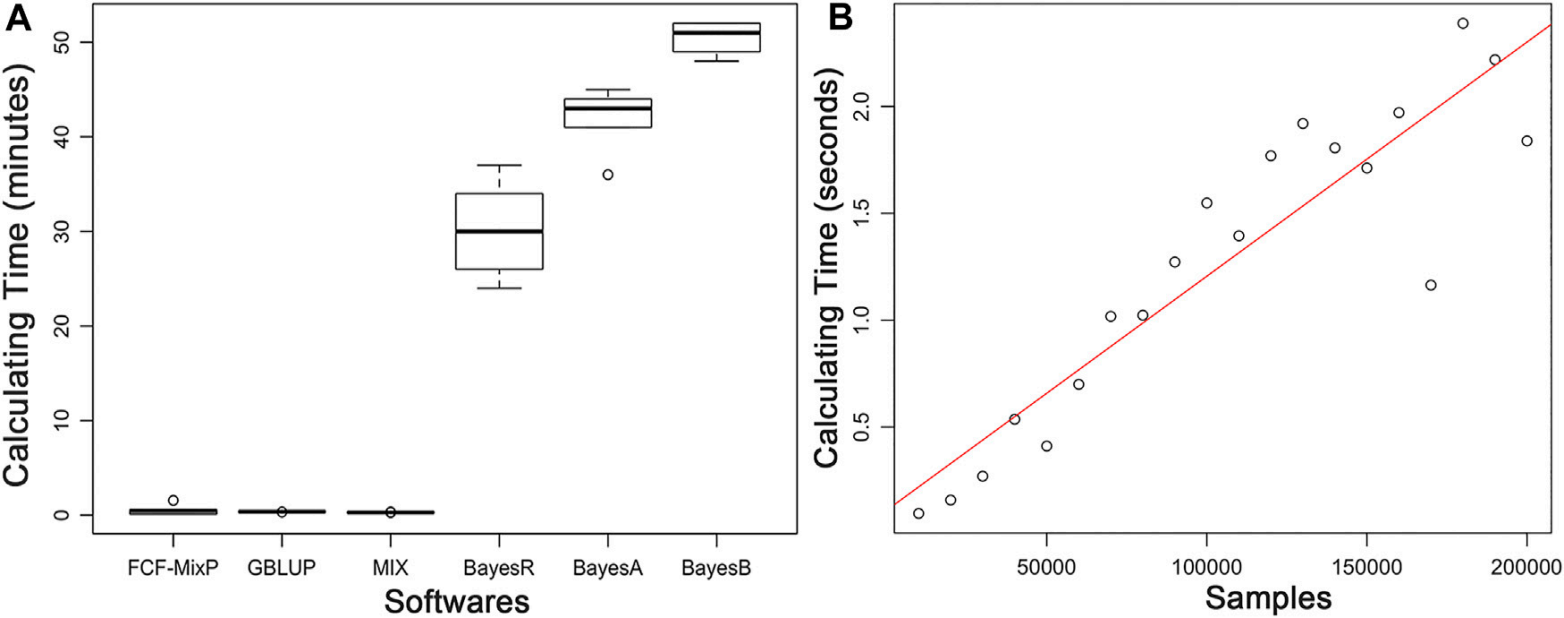
**(A)**. Comparison of computing performances (in min) of FMixFN, GBLUP, MIX, BayesR, BayesA, and BayesB at first cross-validation of the six traits. Computing performance tests were performed in a Red Hat Enterprise Linux server with 2.80 GHz Intel(R) Xeon(R) 20CPUs E5-2680 v4, and 66 GB memory. **(B)**. The computational time (in s) for per iteration of FMixFN according to the number of individuals increasing in the reference group. Computing performance tests were performed on a Red Hat Enterprise Linux server with 2.10 GHz Intel(R) Xeon(R) 64 CPUs Gold 6130, and 131 GB memory.

The predictive ability of each of the six F2 traits under the six predictive methods is shown in Figure 1. For phenotypes 1, 3, and 5, the predictive ability with BayesR, BayesA, and BayesB was, respectively, 0.0377, 0.0308, and 0.0374 higher than that of FMixFN, and the predictive ability of FMixFN was slightly better than of GBLUP by 0.0045, 0.0013, and 0.0103, respectively. For those three phenotypes, there is almost no difference between SSgblup and FMixFN in predictive ability, and FMixFN performs better than SSgblup for phenotype5. For phenotypes 2, 4, and 6, FMixFN performed best for phenotype 4, with a predictive ability 0.0129 higher that of BayesR, BayesA, and BayesB. For phenotype 2, the predictive ability of the five software was similar and was highest with BayesA but only 0.0053 higher than that of FMixFN. For phenotype 6, FMixFN ranked second in the predictive ability, just 0.0027 lower than that of SSgblup. It was worth mentioning that the predictive ability of FMixFN was slightly better than that of other ICE-based Bayesian mixture regression (MIX) by 0.0221, 0.0116, 0.0801, and 0.0263 for phenotype 1, 4, 5, and 6, respectively. The specific information of the predictive ability was also shown in Table 1. Table 2 reports the predictive ability performances of FMixFN and other methods using the Duroc and rice datasets. In the Duroc population, the prediction accuracies were 0.3655, 0.3300, 0.3998, 0.3476, and 0.3589 for FMixFN, GBLUP, BayesR, BayesA, and BayesB, respectively. From the mean value, we found that FMixFN performed slightly worse than BayesR, but outperformed GBLUP, BayesA, and BayesB. In general, the MCMC-based Bayes genome selection algorithm showed some advantages in the traits controlled by several major QTLs, which explained a large proportion of phenotypic variance in some SNPs, while FMixFN performs better than GBLUP. FMixFN is slightly better than some other mainstream methods when traits follow a polygenic model. In this study, we measured the calculation speed of five methods as the average time necessary for the first cross-validation of the six traits. As shown in Figure 2A, the average calculation time was 0.54, 0.37, 0.29, 30.2, 42, and 50.5 min for FMixFN, GBLUP, MIX, BayesR, BayesA, and BayesB, respectively.

**TABLE 1:** Prediction performance in all six traits under the seven predictive methods.

Traits/COR	Methods						
	GBLUP	SSgblup	FMixFN	MIX	BayesR	BayesA	BayesB
phe1	0.661	0.6658	0.6655	0.6434	0.7032	0.6962	0.6977
phe2	0.5637	0.5635	0.5591	0.5602	0.556	0.5564	0.561
phe3	0.4774	0.4803	0.4787	0.4833	0.5051	0.5096	0.509
phe4	0.4814	0.4861	0.4915	0.4799	0.4867	0.4786	0.48
phe5	0.2214	0.2232	0.2317	0.1516	0.2691	0.2485	0.2549
phe6	0.1524	0.1564	0.1537	0.1274	0.1512	0.1529	0.1541
Mean	0.4262	0.4292	0.4300	0.4076	0.4452	0.4403	0.4427

COR: The Pearson correlation coefficient between predicted values and phenotypic value.

**TABLE 2:** Comparison of predictive ability performances of six methods by using Duroc dataset and rice dataset.

Traits/COR	Methods					
	GBLUP	FMixFN	MIX	BayesR	BayesA	BayesB
Duroc	0.33	0.3655	0.3579	0.3998	0.3476	0.3589
FT	0.4347	0.4506	0.4381	0.4415	0.4295	0.4342
CH	0.6000	0.5696	0.5786	0.5830	0.5809	0.5737
Mean	0.4549	0.4619	0.4582	0.4747	0.4526	0.4556

COR: The Pearson correlation coefficient between predicted values and phenotypic value.

### FMixFN When Dealing With Large-Scale Sample Data

The computational time per iteration of FMixFN increases almost linearly as the number of individuals increases in the reference group, as shown in Figure 2B. Data reading time also increased linearly as the amount of sample data increased. Through simulation studies, we also found that FMixFN can calculate GEBV for 200,000 large samples without data overflow. Data overflow usually occurred in exponential functions, where data overflows or underflows could occur when the exponential part of the exponential function was very large or very small. The exponential part of Eq. 5 and Eq. 6 was $\text{exp}\left[-\frac{Y^{2}}{2\left(\delta^{2}+\delta_{i}^{2}\right)}\right]$ , in which $Y={\left(B_{j}^{,}B_{j}\right)}^{-1}B_{j}^{,}y_{-j}$ , $B_{j}$ was the j column of the accompanying matrix of SNPs. When $B_{j}$ growth unlimited, the limit of $\text{exp}\left[-\frac{Y^{2}}{2\left(\delta^{2}+\delta_{i}^{2}\right)}\right]$ is negative infinity, and data underflows will occur. In this study, we found FMixFN could calculate GEBV for 200,000 samples without data overflow. So FMixFN has the potential for use on large-scale sample data.

In conclusion, when there are fewer individuals in the reference group, the computational speeds for ICE-based FMixFN are on the same order of magnitude with GBLUP, and they were much faster than the MCMC-based Bayesian methods. When the number of individuals in the reference dimension of the kinship matrix exceeds hundreds of thousands or even millions, the process to inverse the matrix becomes very difficult for the direct method of genomic selection. FMixFN has excellent computational efficiency and can handle large-scale sample data.

## Discussion

Our results show that the accuracy of genomic selection is affected by many factors, among which the a priori hypothesis on the size of the QTL effect values for traits is crucial. Usually, the most accurate method to predict genetic values or phenotypes based on SNP genotypes is to fit all SNPs simultaneously, treating the SNP effects as they are drawn from a prior distribution that matches the true distribution of SNP effects as closely as possible (Goddard and Hayes, 2009; Chatterjee et al., 2013). To date, the genetic architecture of many traits is still not entirely understood, which means that the prior hypothesis about the QTL effect distribution of all genomic selection models is empirical. In general, mixed normal distributions are more accurate than a single distribution, because the mixture of normal distributions can approximate a wide variety of distributions. It is important to note that this does not imply the SNP effects are drawn from a mixture of normal distributions, but it merely means that such a mixed distribution can approximate almost any distribution that might describe the distribution of effect sizes. BayesR provides an estimate of the number of causal variants that affect a trait and of the distribution of their effects by approximating the distribution of effect sizes with a mixture of normal distributions. In our study, the prior distribution of the SNP effect was similar to BayesR, which came from a mixture of four normal distributions with a ratio of 1:1:1:1 . The difference with BayesR is that we used an Iterative Conditional Expectation (Zhao et al.) algorithm.

Narrow-sense heritability is defined as the proportion of additive variance to phenotype variance (Wray and Visscher, 2008), which means that a trait with a high heritability is more under the control of genes and is less affected by the environment. Therefore, we divided all 374 traits into three groups according to their heritability, and the variance of the mixed distribution is then calculated in each group. The phenotypes included in our study cover almost all the traits measured in pigs and thus are representative, resulting in a high unbiasedness and compatibility variance. This study randomly assumed two sets of variance ratios and used two representative phenotypes to verify the predictive ability but the results showed that the predictive ability obtained using the original variance may not be optimal. The distribution of marker effects for various traits was different, and no one genomic selection model or a priori hypothesis was optimal for all traits. The variance parameters (variance ratios) obtained in our study were expected to be unbiased as the F2 resource population contained a relatively sufficient number of individuals.

The results showed that the predictive ability of BayesR, BayesA, and BayesB was similar in phenotype1, 3, and 5, and was higher than that of the three other methods, which means the MCMC based Bayes genomic selection model has an advantage in predicting genomic breeding values when the trait is affected by large-effect QTL. This result confirms those reported by Chen et al. (2014). For the three phenotypes, GBLUP resulted in the least prediction results because its prior assumption did not match reality as it assumed that traits are controlled by many SNPs, each with a small effect. FMixFN performed slightly better than GBLUP for these three traits, but worse than the Bayes-based methods. Although the prior distribution of FMixFN was a mixture of normal distributions, the posterior variances of SNP effects were not updated, which is a potential drawback for these ICE-based methods. The predictive ability obtained with SSgblup was similar to that with FMixFN because of the addition of pedigree information. However, all the methods yielded almost the same result for phenotypes 2, 4, and 6, a reasonable explanation may be that when the traits are controlled by many polymorphisms of very small effect, the prior hypothesis of the Bayes-based method is closed to that of GBLUP.

In addition to resulting in stable predictive ability, two other advantages of FMixFN are computational efficiency and its ability to deal with large-scaled sample data. The level of the computational efficiency of the direct method of genomic selection was the same as that of FMixFN when the number of individuals in the reference population was small, but if this number increases, the direct method will not be efficient because the process to invert the matrix will become very difficult due to the limitations in computer memory and computational time. Our study demonstrates the stability of FMixFN and its potential for use on large-scale sample data.

## Conclusion

We have developed a Bayes-based genomic selection model called FMixFN, which combines stable predictive ability and computational efficiency. Besides, when the number of individuals in the reference population is large, FMixFN is one of the best choices for genomic selection. FMixFN is a stable, big data-oriented genomic selection model, which could meet the needs of large breeding companies or combined breeding schedules.

## Data Availability

The datasets presented in this study can be found in online repositories. The names of the repository/repositories and accession number(s) can be found in the article/Supplementary Material.
