# Supplementary material for: FMixFN: A Fast Big Data-Oriented Genomic Selection Model Based on an Iterative Conditional Expectation algorithm
Source: Front Genet. 2021 Nov 18;12:721600. doi: 10.3389/fgene.2021.721600 (PMC8637923; doi:10.3389/fgene.2021.721600)
Supplement: Supplementary file 1 [file DataSheet2.doc]

**
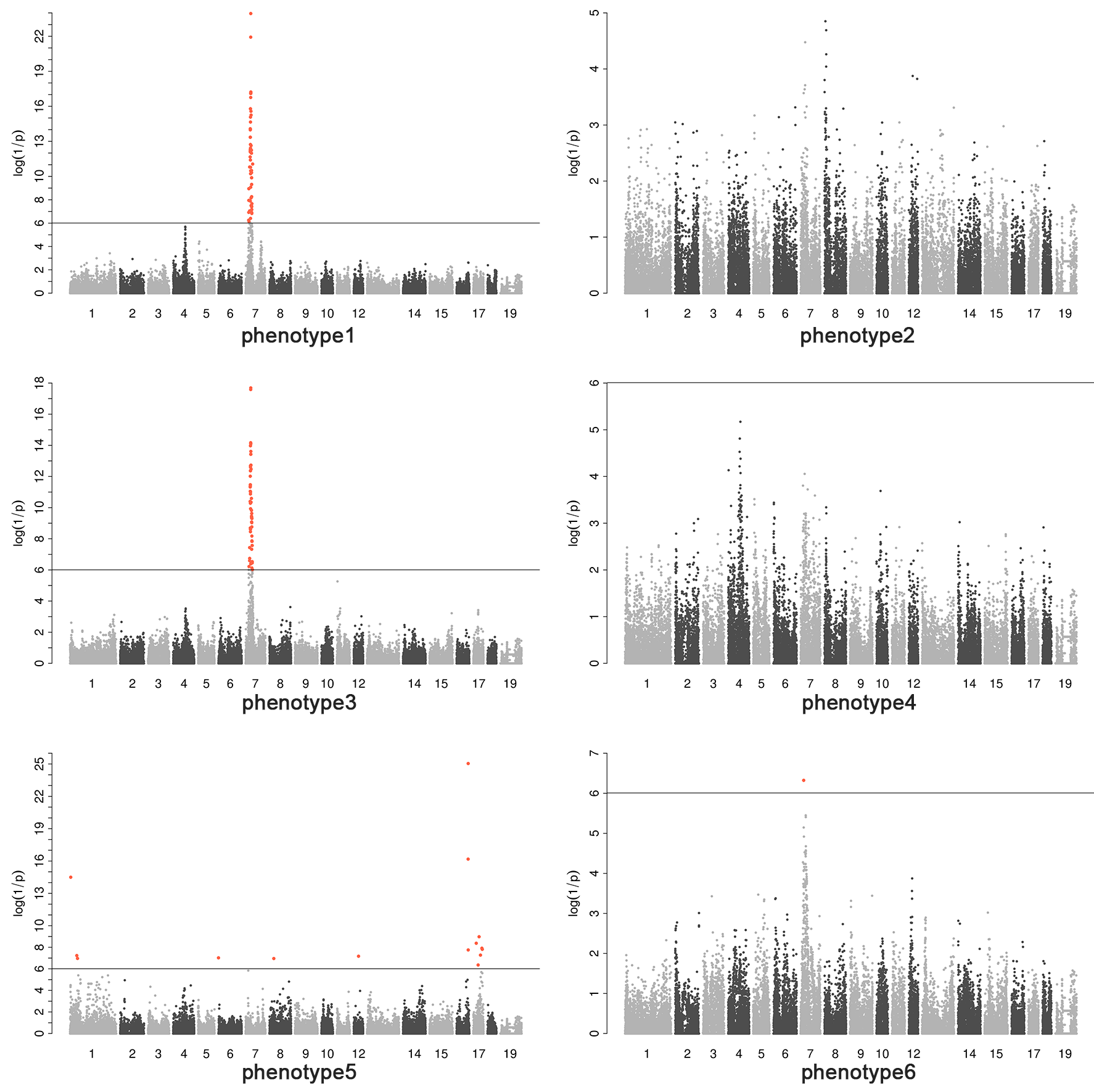
**

**Figure S1 GWAS results for six phenotypes.** The y-axis and the x-axis represent the negative $log10$ P-value of the SNPs and the genomic positions separated by chromosomes, respectively, and the black solid lines indicate the significance threshold.
